# Supplementary material for: Elovl2 Is Required for Robust Visual Function in Zebrafish
Source: Cells. 2020 Dec 2;9(12):2583. doi: 10.3390/cells9122583 (PMC7761535; doi:10.3390/cells9122583)
Supplement: Supplementary file 1 [file cells-09-02583-s001.pdf]

Supplementary figures

A

Organism	Elovl2 % protein sequence conservation
Macaca mulatta	99.0%
Mus musculus	88.5%
Gallus gallus	78.1%
Danio rerio	65.2%

B

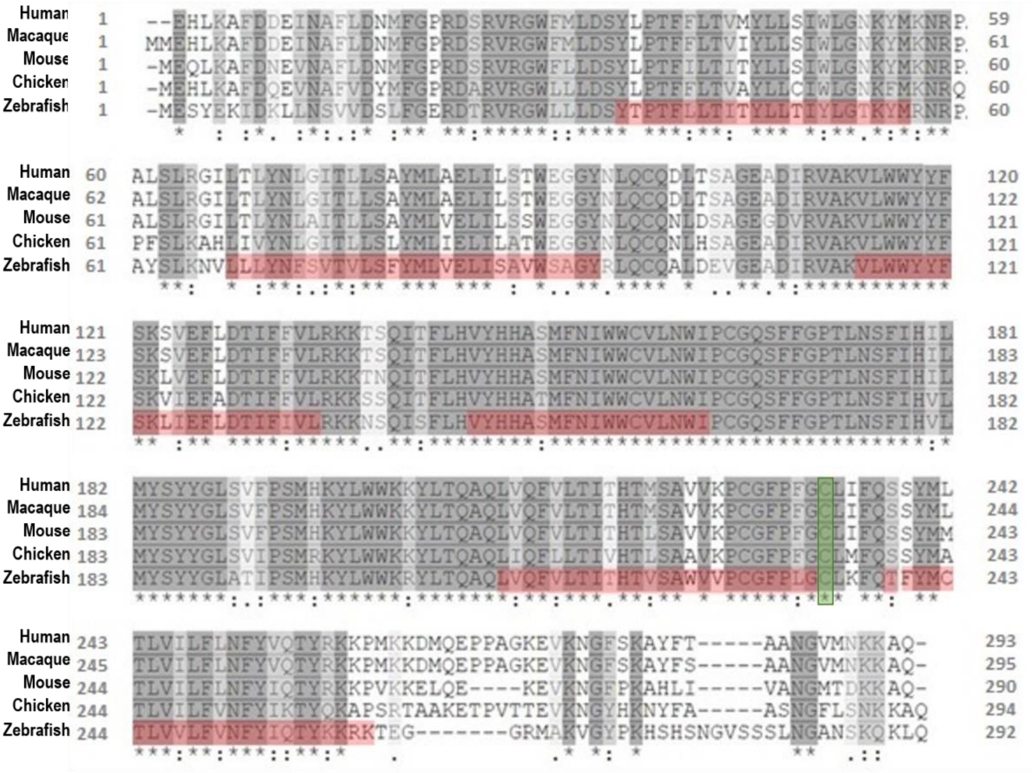

Supplementary Fig 1

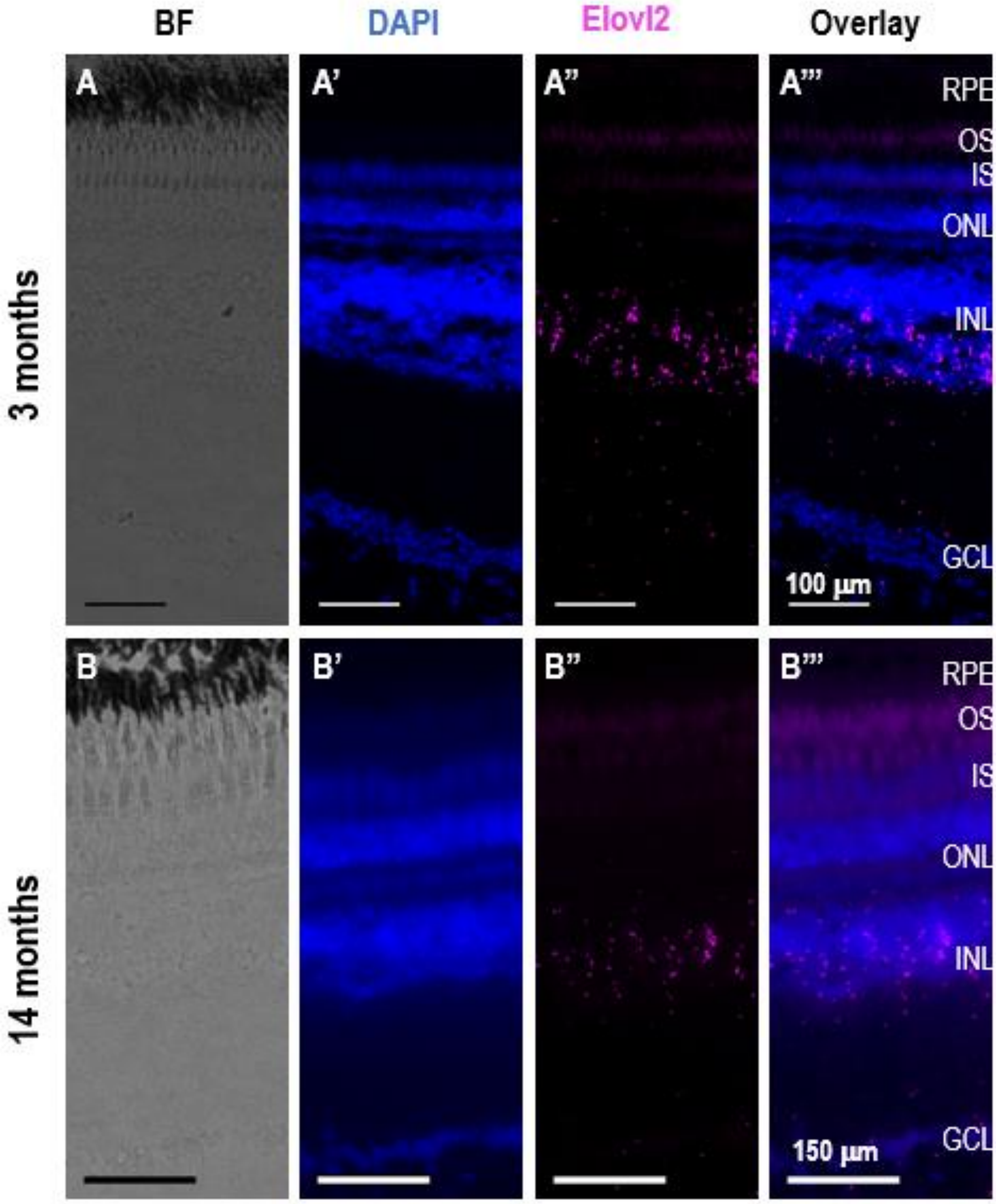

Supplementary Fig 2

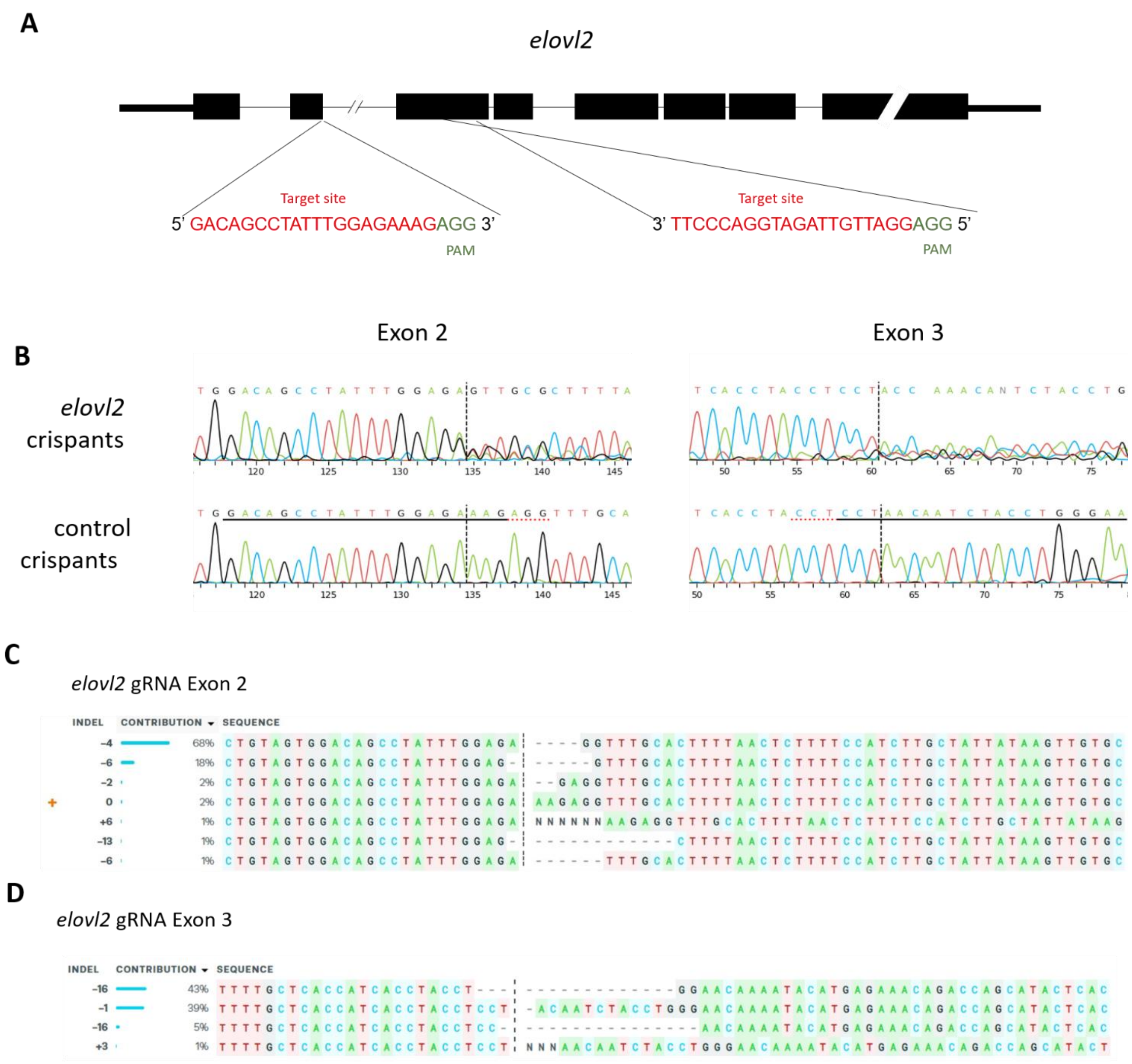

Supplementary Fig 3

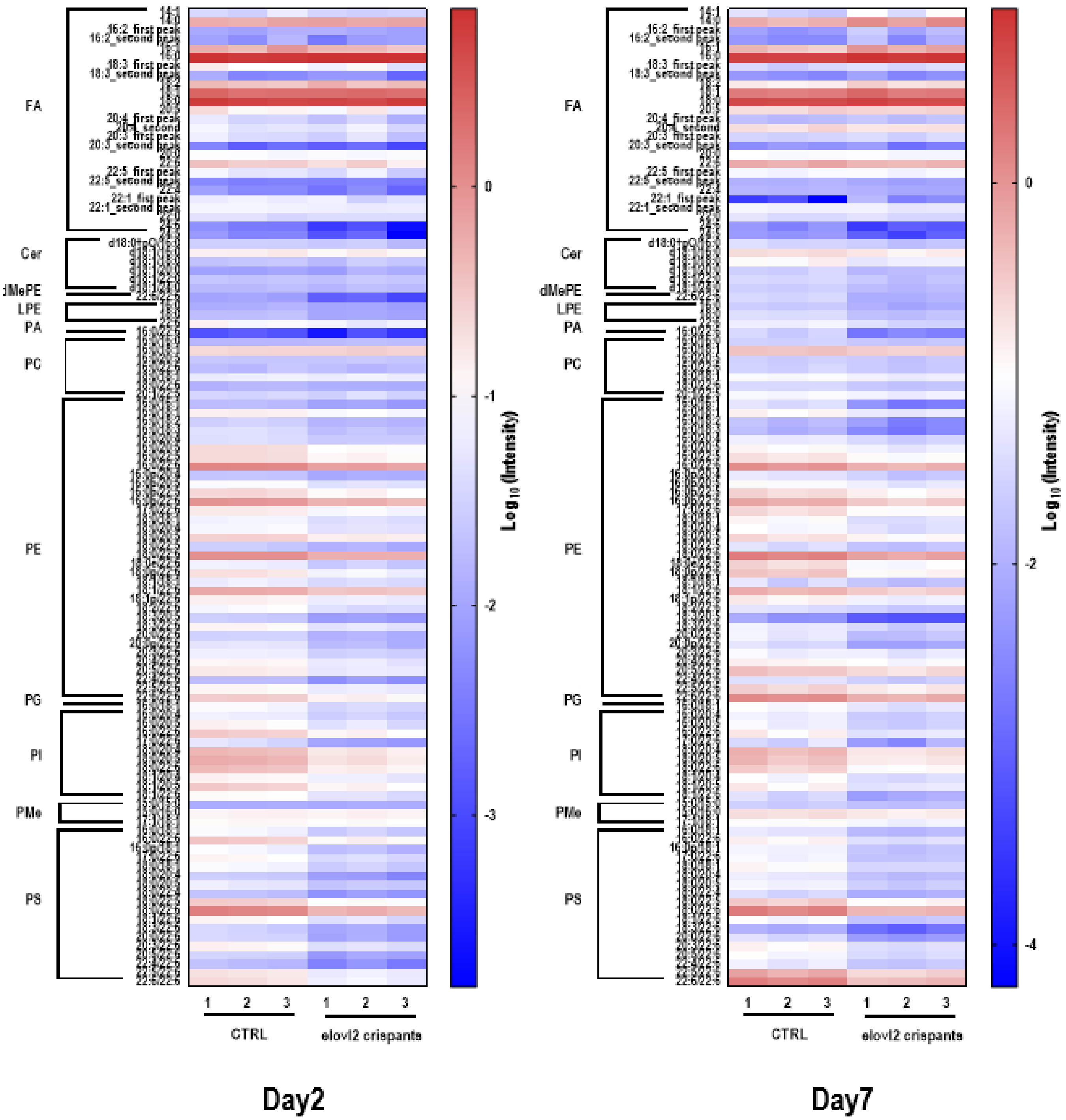

Supplementary Fig 4

Day2

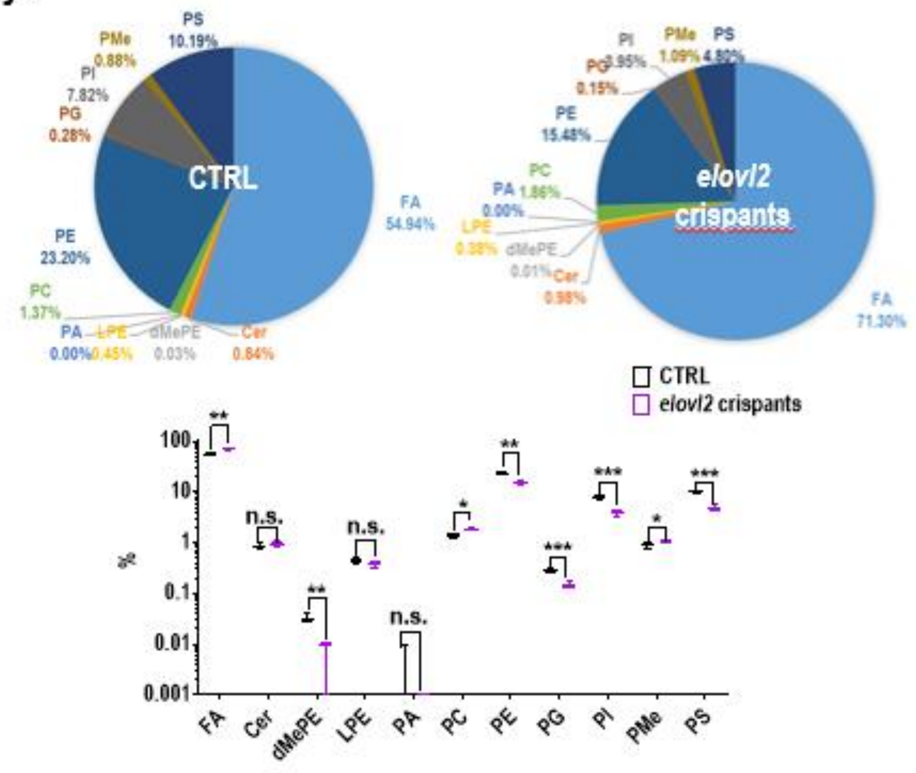

Day7

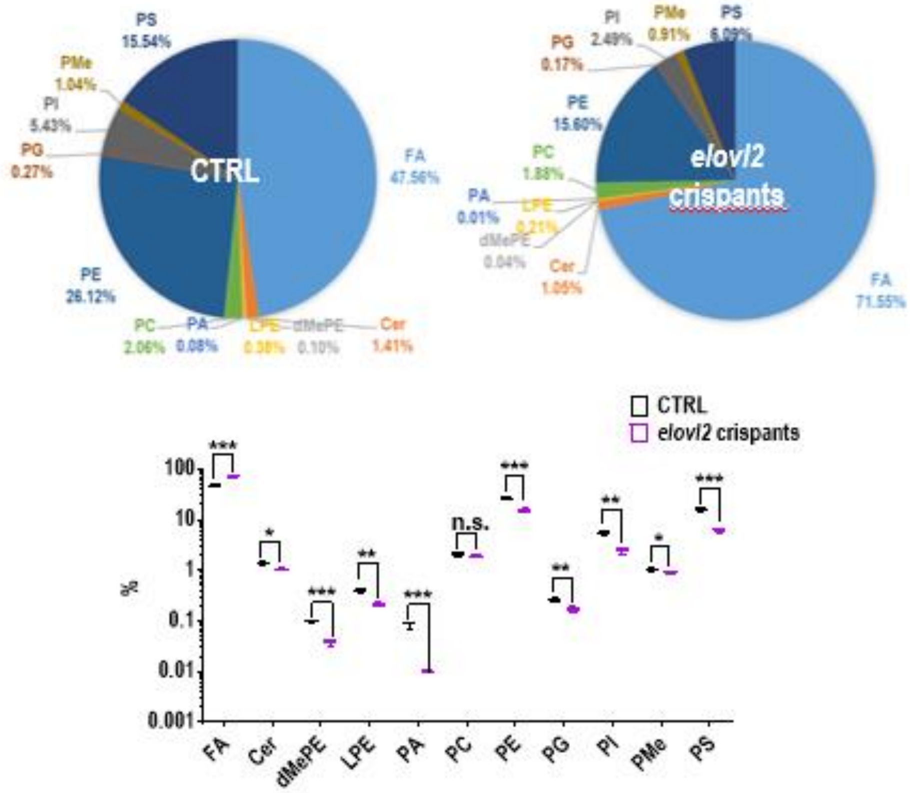

Supplementary Fig 5

Supplementary Fig 6

A

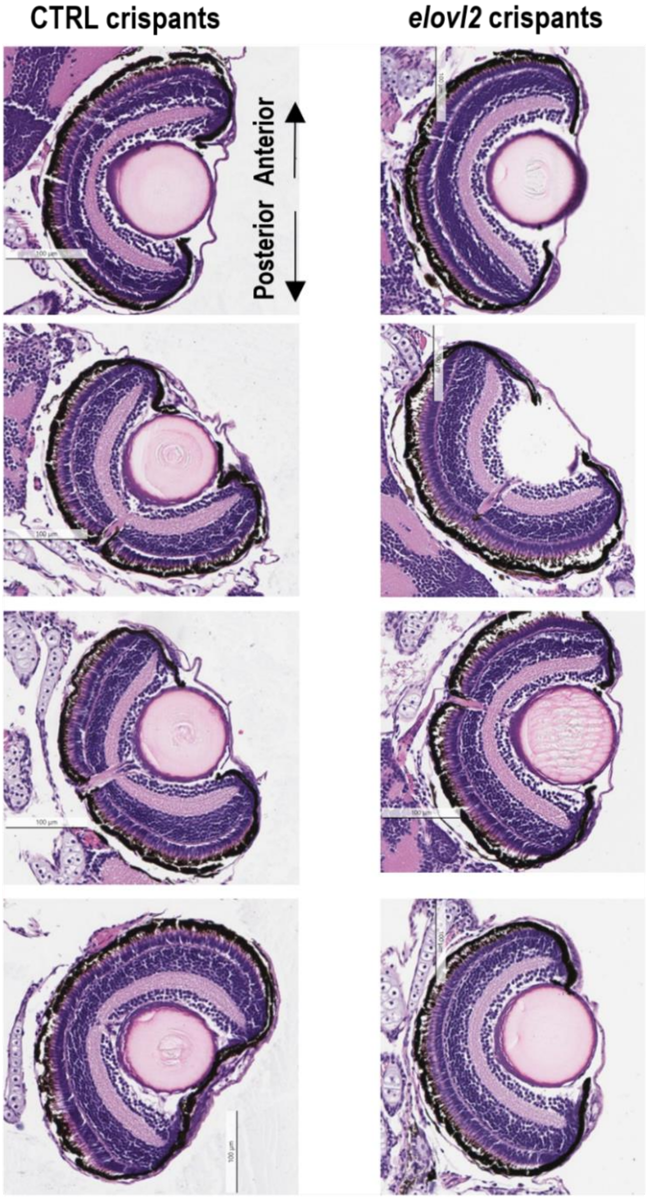

B

	Anterior	Middle	Posterior
RGL	2.968 ± 0.651 (21.42 ± 4.7%)	3.62 ± 0.749 (27.97 ± 5.79%)	2.1 ± 0.7 (13.28 ± 4.43%)
IPL	1.888 ± 0.489 (14.61 ± 3.78%)	2.03 ± 0.55 (17.94 ± 4.85%)	n.s.
INL	3.643 ± 0.51 (17.8 ± 2.47%)	3.79 ± 0.53 (20.75 ± 2.9%)	2.256 ± 0.62 (9.96 ± 2.73%)
ONL	n.s.	1.34 ± 0.55 (18.89 ± 7.7%)	1.008 ± 0.447 (12.14 ± 5.38%)
PRL	n.s	2.532 ± 0.621 (15.26 ± 3.74%)	3.986 ± 0.697 (24.53 ± 4.29%)

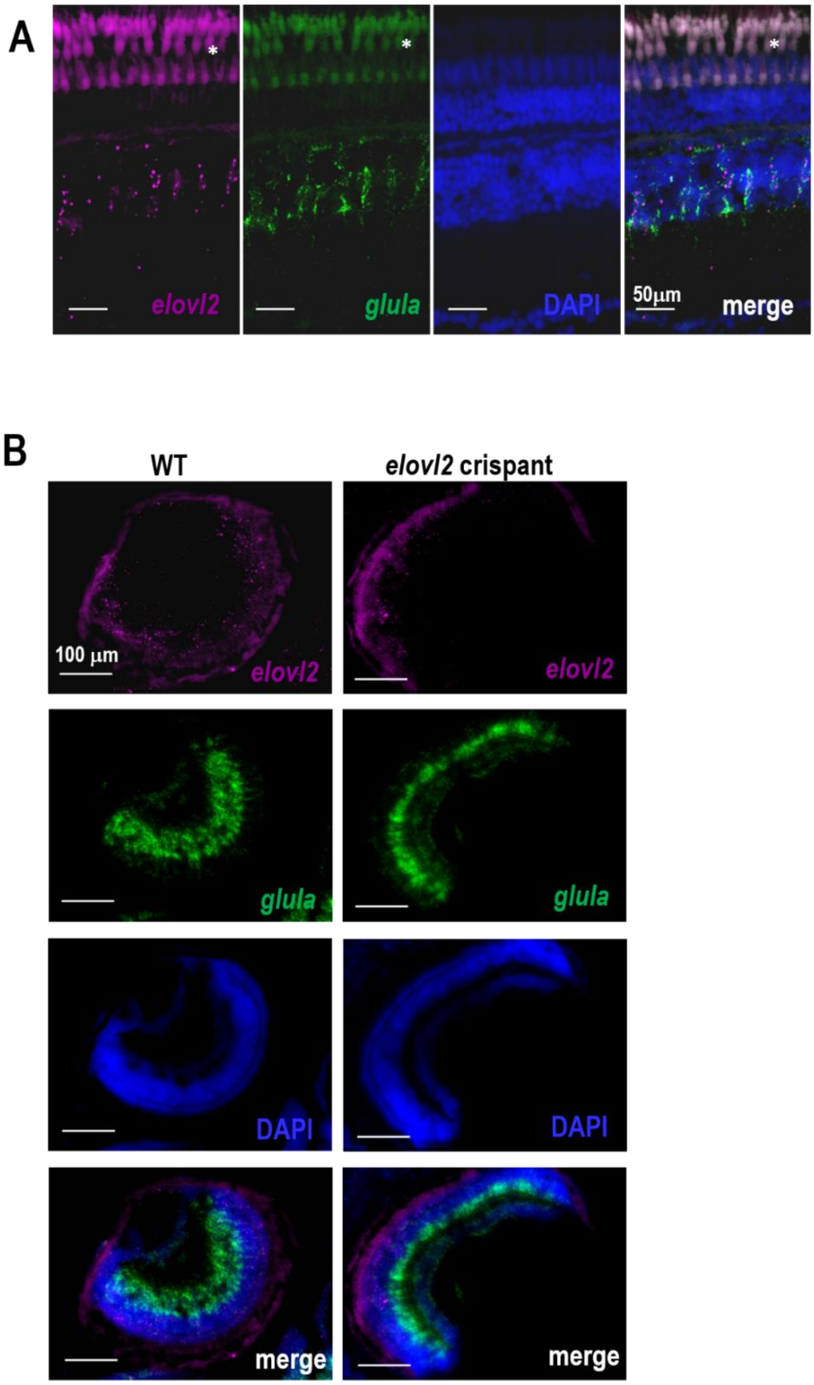

Supplementary Fig 7

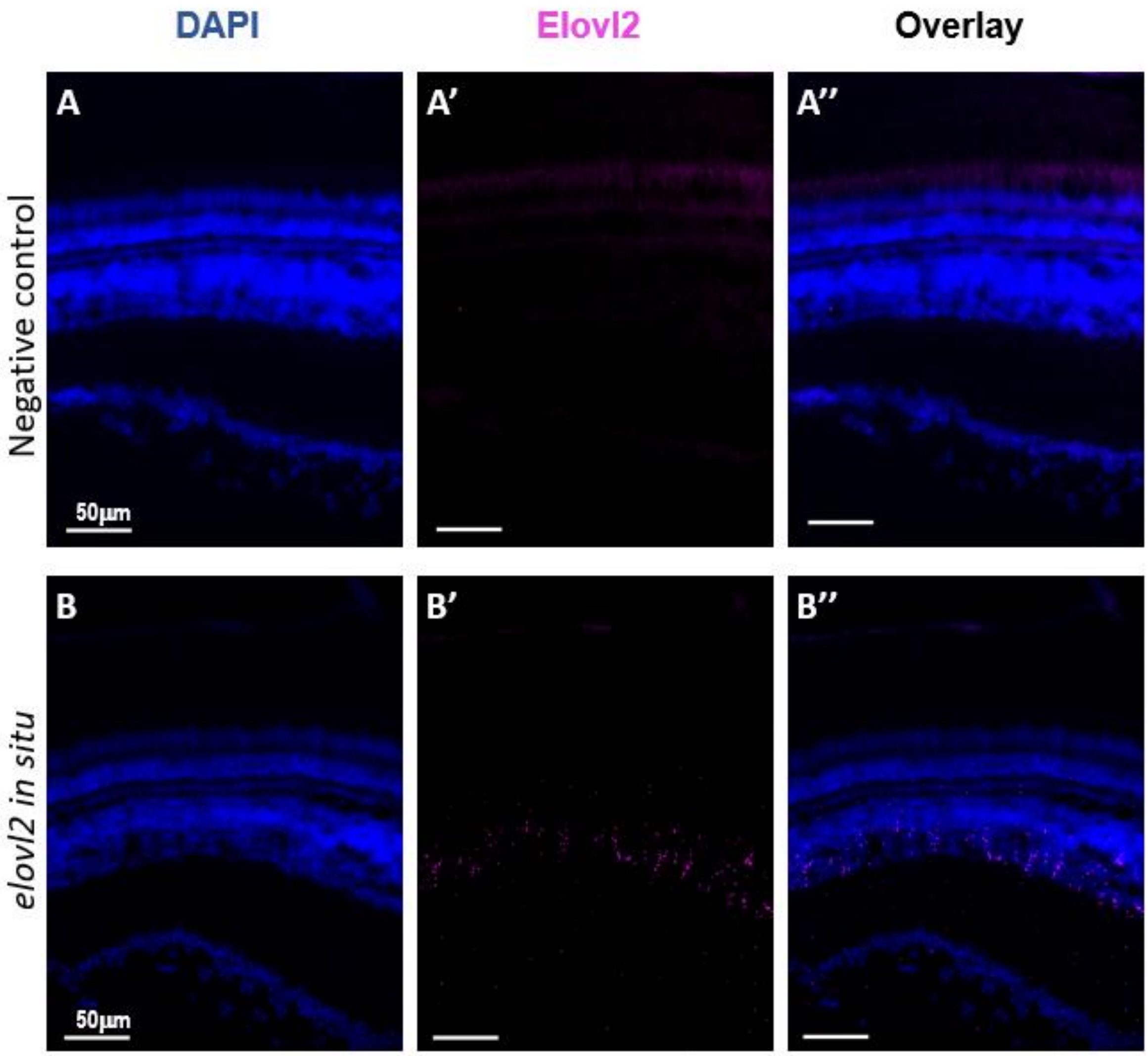

Supplementary Fig 8
